# Synthetic nanocomposite MgH_2_/5 wt. % TiMn_2_ powders for solid-hydrogen storage tank integrated with PEM fuel cell

**DOI:** 10.1038/s41598-017-13483-0

**Published:** 2017-10-16

**Authors:** M. Sherif El-Eskandarany, Ehab Shaban, Fahad Aldakheel, Abdullah Alkandary, Montaha Behbehani, M. Al-Saidi

**Affiliations:** 1 Nanotechnology and Advanced Materials Program, Energy and Building Research Center, Kuwait Institute for Scientific Research, Safat, 13109 Kuwait, Kuwait; 2 Environment Pollution and Climate Program, Environment and Life Sciences Research Center, Kuwait Institute for Scientific Research, Safat, 13109 Kuwait, Kuwait

## Abstract

Storing hydrogen gas into cylinders under high pressure of 350 bar is not safe and still needs many intensive studies dedic ated for tank’s manufacturing. Liquid hydrogen faces also severe practical difficulties due to its very low density, leading to larger fuel tanks three times larger than traditional gasoline tank. Moreover, converting hydrogen gas into liquid phase is not an economic process since it consumes high energy needed to cool down the gas temperature to −252.8 °C. One practical solution is storing hydrogen gas in metal lattice such as Mg powder and its nanocomposites in the form of MgH_2_. There are two major issues should be solved first. One related to MgH_2_ in which its inherent poor hydrogenation/dehydrogenation kinetics and high thermal stability must be improved. Secondly, related to providing a safe tank. Here we have succeeded to prepare a new binary system of MgH_2_/5 wt. % TiMn_2_ nanocomposite powder that show excellent hydrogenation/dehydrogenation behavior at relatively low temperature (250 °C) with long cycle-life-time (1400 h). Moreover, a simple hydrogen storage tank filled with our synthetic nanocomposite powders was designed and tested in electrical charging a battery of a cell phone device at 180 °C through a commercial fuel cell.

## Introduction

Hydrogen is an energy carrier, which holds tremendous promise as a new clean energy option^1,2^. It is a convenient, safe, versatile fuel source that can be easily converted to a desired form of energy without releasing harmful emissions^3,4^. A key advantage of hydrogen is that when burned, carbon dioxide (CO_2_) is not produced. Hydrogen storage is one of the most crucial difficulties restricting utilization of hydrogen energy for real applications. However, storage hydrogen gas into gas cylinders under high pressure reached to 350 bar is a well-known technology, using such pressurized hydrogen gas tanks as a source of fuel in vehicles is not safe at present and still needs many intensive studies dedicated for improving the structural and mechanical properties of the materials used in tank’s manufacturing. Extreme conditions for on-road vehicle service should be defined, to demonstrate performance of storage systems demonstrated both under the stresses of normal vehicle operation and under externally imposed stresses^5^. Likewise pressurized hydrogen gas, liquid nitrogen possesses many difficulties related to its very low density. Accordingly, the size of liquid hydrogen requires larger tanks reaches to about three times larger than the traditional gasoline tank^6^. Practically, converting hydrogen gas into liquid hydrogen is not an economic process since it consumes large amount of energy required to cool down the gas temperature to −252.8 °C. For instance, liquefying 1 kg of hydrogen gas in medium-size plant requires 10 to 13 kWh of electrical energy^7^. Moreover, liquid nitrogen is not safe since it has a high flammability range. Boil-off losses associated with the storage, transportation and handling of liquid nitrogen can consume up to 40% of its available combustion energy^6,8^.

## Solid Hydrogen

Apart from gaseous and liquidus phases of hydrogen, solid hydrogen has been considered as the most reliable and safe practical solution for providing clean energy required for different applications, using proper fuel cells such as proton-exchange fuel cells membrane (PEM)^9^. Hydrogen can be simply stored in nanocrystalline metal powders such as Mg and Mg-based nanocomposite powders in the form of MgH_2_. The choice of Mg is attributed to its high hydrogen capacity (7.60 wt. %, 0.11 kg H_2_L^−1^), natural abundance, cheap price, operational cost effectiveness, and light weight. Accordingly, MgH_2_ has become a potential candidate for fuel cell applications used in light-duty vehicles and mobile application^10^.

However, nanotechnology has had an obvious impact on producing industrial scale of uniform nanocrystalline MgH_2_ powders, using a room-temperature reactive ball milling technique (RBM)^10,11^, the nanophase of such metal hydride system still shows serious drawbacks that should be solved first before nominating the system for real applications. Firstly, MgH_2_ has a high thermal stability making the hydrogen releasing at moderate temperatures (below 300 °C) very difficult^2,12,13^. Secondly, MgH_2_ exhibits very slow kinetics of hydrogenation/dehydrogenation at temperatures around 350 °C^14,15^. Innumerable efforts have been tackled to improve the kinetics behavior of MgH_2_ by catalyzing the metal hydride powders with wide spectrum of mono, binary and multicatalytic systems. One of the earliest work proposed for improve MgH_2_ powders was achieved by Prof. R. Schulz and his team work in 1999^16^. In their work, MgH_2_ powders were catalyzed by ball milling with one of 3-d transition metal powders of Ti, V, Mn, Fe and Ni. Based on their results, Ti and V showed better catalytic effect for hydrogen absorption and desorption when compared with Ni. Furthermore, Hanada *et al*.^17^ reported very interesting results on catalyzing of MgH_2_ powders by small amount (1 mol. %) of Fe, Co, Ni and Cu nanoparticles. The as-mechanically doped MgH_2_/Ni powders obtained after a very short milling time (2 h) showed excellent hydrogenation/dehydrogenation kinetics properties and enjoyed high storage capacity (~6.5 wt.%)^17^. Since then, different schools have reported attractive results upon using pure elemental powders such as Al, Ti, Fe, Ni, Cu and Nb^18^, intermetallic compounds^19,20^, metastable big-cube Zr_2_Ni^21^, and metal/metal oxide binary nanocomposite^22^ for improving the kinetics of hydrogen absorption/desorption of MgH_2_. More recently, an interesting study was reported by Ouyang *et al*.^23^ when they successfully prepared Mg_2_In_0.1_Ni solid solution with an Mg_2_Ni-type structure. They pointed out that the introduction of In-semimetal into Mg_2_Ni not only significantly improved the dehydrogenation kinetics but also greatly lowered the thermodynamic stability. This was implied by lowering the activation energy of dehydrogenation and enthalpy change to very low values of 28.9 kJ/mol and 38.4 kJ/mol H_2_, respectively^23^.

Besides metal, semimetal, and metallic metastable phases, hard metal oxide of Nb_2_O_5_^24^ and refractory material powders and, such as SiC^25^, and TiC^26^ find a space of applications as excellent kinetics modifier used successfully for improving the hydrogen absorption/desorption behaviors of MgH_2_ system. More recently, we demonstrated the first report of employing a metallic glassy Zr_70_Ni_20_Pd_10_ powders for enhancing the hydrogenation/dehydrogenation properties of MgH_2_ powders^4^. In general, it is agreed that mechanically-induced doping of MgH_2_ with the abrasive powders of hard phases such as carbides, oxides, intermetallic and metallic glassy alloys materials lead to fast grain refining of the MgH_2_ upon releasing the crystalline stored energy, leading to refine the MgH_2_ grains along their grain boundaries where superfine grains are formed. Such desirable fine grains with their short-distance grain boundaries always facilitate short diffusion path^22^, leading to fast diffusion of the hydrogen atoms^26–28^. Recently, Crivello *et al*.^29^ have introduced a useful review article discussing the several ways used for improving MgH_2_-based materials.

Recently, Zhu *et al*. investigated a powerful technique called plasma-milling (P-milling)^30^ when they successfully introduced dielectric barrier discharge plasma (DBDP) during vibratory milling of Mg(In)-MgF_2_ composite powders^31^. The combination between the plasma process, and the impact and shear forces generated by the milling balls led to enhance the milling process for refining and/or alloying the milled powders^32^.

Apart from the milling process employed for preparing MgH_2_ nanocrystalline and MgH_2_-based nanocomposite powders, direct current magnetron sputtering technique was successfully employed in 2004 by Ouyang *et al*.^33^ for preparing multi-layer hydrogen storage thin films with Mg and MmNi_3.5_(CoAlMn)_1.5_. They reported that this technique can led to the formation of high quality nano-scale multi-layer composite with well-controlled film thickness and well-bonded interface between the multilayers^33^. One advantage of this promising technique can be realized from its ability on synthesizing ultra-thin films (4 nm) with clean interfaces.

## Solid Hydrogen Tanks

Merits of using solid hydrogen tanks are obvious for those who live in isolated communities having lack in connection to the public power grid. However, renewable energy sources that generate electricity directly through for example photovoltaics (PVs) and windmills are environmentally friendly and can provide cost-effective solutions, their energy availability varies drastically from time to day per one day. Accordingly, energy storage is necessary to meet the electricity demand with the required reliability^34^. Thus, the combination of stored solid hydrogen generated from excess electricity and a fuel cell is a promising solution^35^.

In general solid hydrogen storage tanks differ in their design and materials used when compared with those used to store pressurized hydrogen gas and liquid hydrogen. This because diffusion of hydrogen atoms into metal lattice (e.g. Mg) does not require the application of very high pressure since the gas-solid exothermic reaction between the two phases takes place simultaneously at relatively low pressure (below 15 bar/300 °C). Moreover, the need of using expensive cryotanks with controlled pressure (similar to those used for liquid hydrogen) is not required. Accordingly, the basic process for design, materials selections, and manufacturing of vessels or tanks contain metal hydride, as a source of hydrogen becomes inexpensive in materials science point of view. In fact, the success of using solid hydrogen tank for providing hydrogen to operate an electrical system with constant rate of hydrogen flow through a suitable fuel cell can be firstly attributed to the hydrogenation/dehydrogenation kinetics behavior and cyclability of the metal hydride materials stored in the tank.

Most the authors published fine articles related to the Mg- based hydrogen storage materials, focused mainly on materials preparations, characterization and investigation the hydrogenation/dehydrogenation behaviors of the metal hydride systems. However, there are some studies reported interesting and promising results related to designing and utilizing of solid hydrogen tank. Of these, Zu *et al*.^36^ proposed an advanced design that can be used for real manufacturing of hydrogen storage tank. However, their design may improve the geometric flexibility and structural performance of composite toroidal hydrogen storage tanks; they have not examined the system with any types of metal hydride to ensure the validity of their tank model for hydrogenation/dehydrogenation processes. A numerical work proposed by Gkanas *et al*.^37^ showed a three-dimensional computational model regarding coupled heat and mass transfer during both the hydrogenation and dehydrogenation process. In this study, two different types of hydrides, LaNi_5_ and an AB2-type (Ti-Zr-Mn intermetallic system) were selected as possible candidate materials. Their results showed the possibility of hydrogen gas uptake and release with moderate kinetics at 20 °C and 15 bar of H_2_. A very interesting and promising model was recently introduced by Gattia *et al*.^38^. They used compacted powders prepared by ball milling MgH_2_ with Nb_2_O_5_ with mixed with 5 wt.% ENG. The pellets were coated by different type of metal coating, using sputtering and thermal evaporation techniques. The material were tested by inserting the pellets into a cylindrical tank. Unfortunately, the kinetics of the system did not show attractive characteristics since the temperature required for cyclic procedure taken place at 310 °C.

On the other hand, significant number of patents related to designing hydrogen storage tanks are available. One of the earliest invention related to this regard dates back to 1969, when Lyon *et al*. proposed a process and an apparatus used to utilize MgH_2_ as a source of hydrogen for useful fuel cell applications^39^. In their prototype, Mg reacted with hydrogen gas to form MgH_2_ and then decomposed at high temperature (277 °C to 649 °C) under pressure ranging from 1 bar to 207 bar^39^. Applications of such high temperatures and pressure are consider as drawback of their system. In 2014, Ornath^40^, introduced an interesting idea for manufacturing a hydrogen storage tank that can lead to lower the temperature of hydrogen uptake and release. Unluckily his patent did not contain any experimental results to prove his idea. Details and more information on the employing different types of metal hydride materials on solid-state hydrogen storage are recently reviewed and published by Rusman and Dahari *et al*.^41^.

Apart from the interesting and useful solid hydrogen storage models invented by many authors, our present has been addressed to satisfy two objectives; the first is focused on synthesizing and characterizing a new nanocomposite MgH_2_/5 wt. % TiMn_2_ system with advanced kinetics behaviors and cyclability, where the second objective is focused on utilizing the as-synthesized nanocomposite powders in a self-manufactured hydrogen storage tank interfaced to 40 W/4.5 A proton exchange membrane (PEM) fuel cell. This system feeds the PEM-fuel cell with the released hydrogen gas form the tank. The converted electrical energy was utilized to charge the battery of a cell phone device through 5 V voltage regulator. As far as the authors know, this is the first time to examine the validity of a new Mg-based nanocomposite in real applications.

## Results and Discussion

### Structure

**Figure 1:**
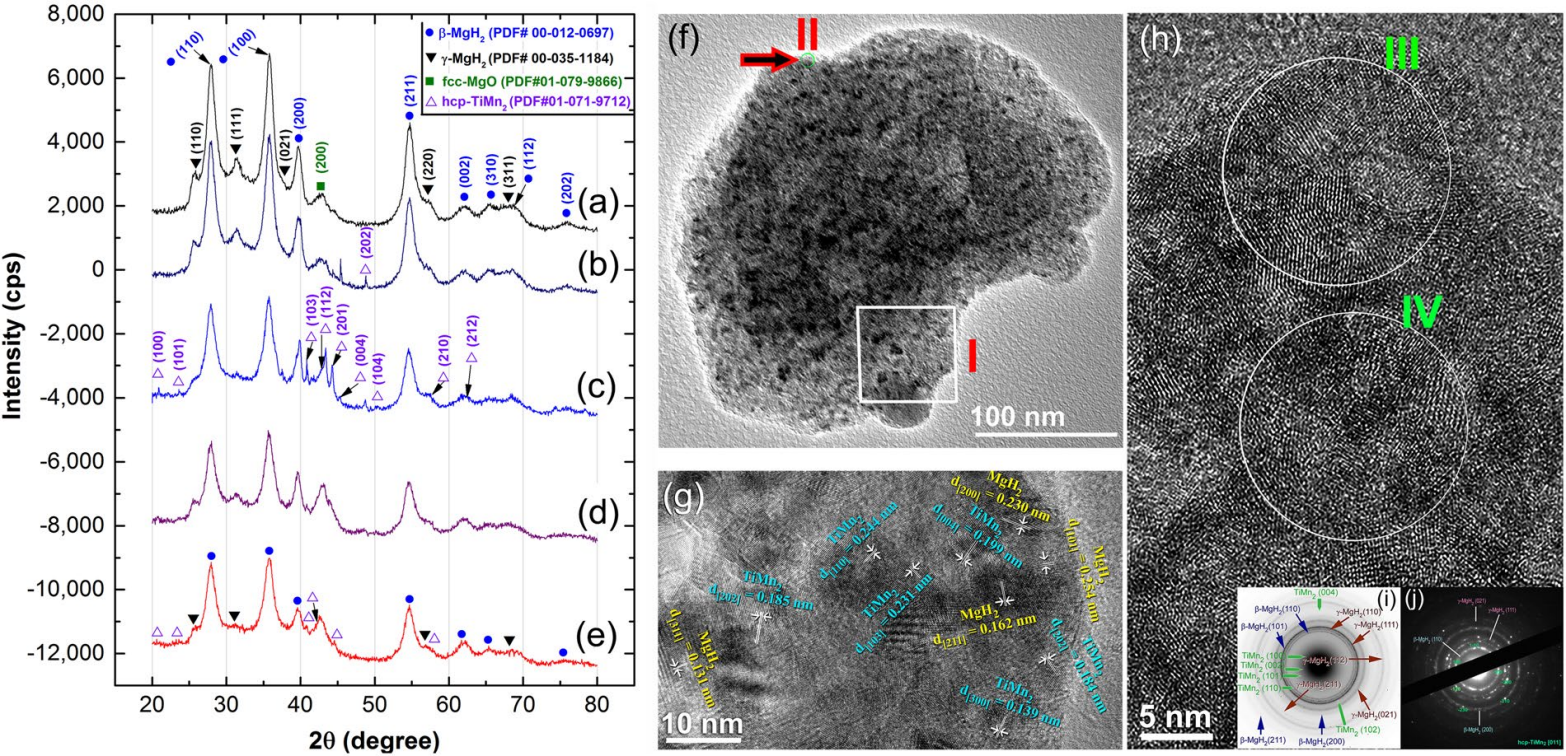
Structural characteristics of nanocrystalline MgH_2_ and nanocomposite MgH_2_/5 wt.% TiMn_2_ powders milled for different RBM time. (**a**) XRD patterns of hcp-Mg powder obtained after ball milling under 50 bar of H_2_ gas atmosphere for 200 h. XRD patterns of as-synthesized MgH_2_ powder doped with 5 wt.% TiMn_2_ powder and then ball milled for (**b**) 3 h, (**c**) 12.5 h, (**d**) 37.5 h, and (**e**) 50 h. The BFI image of the end-product (50 h) is shown in (**f**), where the HRTEM image for zone I indexed in (**f**) is presented in (**g**). HRTEM image of zone II indexed in (**f**) is presented in (**h**). The NBDPs for zones III and IV shown in (**h**) are elucidated in (**i**,**j**), respectively.

The general and local structure beyond the nano level for the nanocomposite MgH_2_/5 wt. % TiMn_2_ powders were investigated by means of X-ray diffraction (XRD) and field emission-high resolution transmission electron microscope (FE-HRTEM) techniques, respectively. The XRD pattern of elemental Mg powders (precursor) obtained after 200 h of RBM under 50 bar of a hydrogen gas atmosphere is shown in Fig. 1(a). The powders consisted of fine nanocrystalline mixture of β-MgH_2_ (PDF file# 00-012-0697) and γ-MgH_2_ (PDF file# 00-035-1184) phases, implied by the broad Bragg peaks shown in Fig. 1(a). Low intensity Bragg peak related to fcc-MgO (PDF file# 01-079-9866) was detected at scanning angle of about 42° due to the oxidation of the sample during XRD sample preparation outside of the glove box.

Based on the purpose of the present study, the end-product (200 h) of as-synthesized MgH_2_ powders was manually doped with 5 wt. % TiMn_2_ powders inside the glove box and then charged into the milling vial together with the milling media. The vial was then pressurized with 50 bar of hydrogen gas and mountained onto a high-energy ball mill where the RBM process taking place for different milling time. After 3 h of milling, the powders revealed Bragg peaks related to MgH_2_ phase, where the Bragg peaks corresponding to TiMn_2_ powders were hardly seen, as shown in Fig. 1(b). This may be attributed to the effect of cold welding generated by the milling process on TiMn_2_ powders, leading to sticking the powders onto the milling media (vial’s internal wall and balls). Further ball milling time led to fragmentation of TiMn_2_ agglomerate powders to fall into the vial’s milling zone and then milled together with MgH_2_ powders. After 12.5 h, the milled powders consisted of ultrafine particles of MgH_2_ coexisted with TiMn_2_ (PDF file# 01-071-9712) powders, as implied by the broad Bragg peaks related to both phases (Fig. 1(c)). This broadening manifested in the Bragg peaks raised from both refinement of the MgH_2_ and TiMn_2_ crystallites and accumulated macrostrain during the RBM process. It should be notified that neither reacted powders related to the formation MgTiH_x_, and TiH_2_^42^ phases nor elemental phases corresponding to metallic Mg and Ti could be detected even after longer RBM time, ranging between 37.5 h (Fig. 1(d)) and 50 h (Fig. 1(e)). Moreover, the Bragg-peaks of metallic hcp-TiMn_2_ maintained their peak positions after 50 h of RBM, implying the absence of solubility into the MgH_2_ lattice, as elucidated in Fig. 1(e).

The bright field image (BFI) of nanocomposite MgH_2_/5 wt.% TiMn_2_ powders obtained after 50 h of RBM time is displayed in Fig. 1(f). The nanopowers obtained after this stage of milling were aggregated to form larger particles due to van der Waals forces^43^ containing fine nano-dimensional dark-grey lenses related to TiMn_2_ phase embedded into the light-grey MgH_2_ matrix, as shown in Fig. 1(f). The FE-HRTEM image of zone I indexed in Fig. 1(f) is displayed in Fig. 1(g). The nanocomposite powders revealed Moiré-like fringes with nanocrystalline-structure morphology (5 nm to 17 nm). Moreover, the lattice fringes of MgH_2_ (β and γ phases) and hcp-TiMn_2_ were regularly separated with an interplanar spacing (d) matching well with the reported PDF files cited in Fig. 1(a). Based on careful analysis performed of at least 30 examined zones for three individual samples, we could not detect the existence of any other phase(s). This implies the formation of binary nanocomposite MgH_2_/5 wt.% TiMn_2_ powders. A different examined zone located at the top edge of the particle, zone (II) was analyzed by HRTEM (Fig. 1(h)). Two selected zones (III, and IV) in Fig. 1(h) were selected to get their corresponding nano beam diffraction patterns (NBDPs). The grains located in each zone contained ultrafine nano-grains ranging in size between 5 to 8 nm, as displayed in Fig. 1(h). It can be notified that the grains did not reveal specific orientation (Fig. 1(h)) and the development of dislocation tangles into sub-boundaries can be seen clearly in zones III and IV (Fig. 1(h)). The dislocations presented inside the individual grains suggesting the nanostructure development by severe plastic deformation created by the ball milling media.

The NBDPs taken from the zone III and IV elucidated in Fig. 1(h) are shown in Fig. 1(i) and (j), respectively. Both electron diffraction revealed Debye continuous rings corresponding to β- and γ-MgH_2_ phases, plus hcp-TiMn_2_. The present of these diffracted rings indicates the random orientation of the grains in the nanocomposite powders (Fig. 1(i)). In addition, the circular-like NBDPs of MgH_2_ (Fig. 1(j)) were overlapped with numerous closely spaced spots, each from diffraction of a single crystallite for TiMn_2_.

### Morphology and elemental analysis

**Figure 2:**
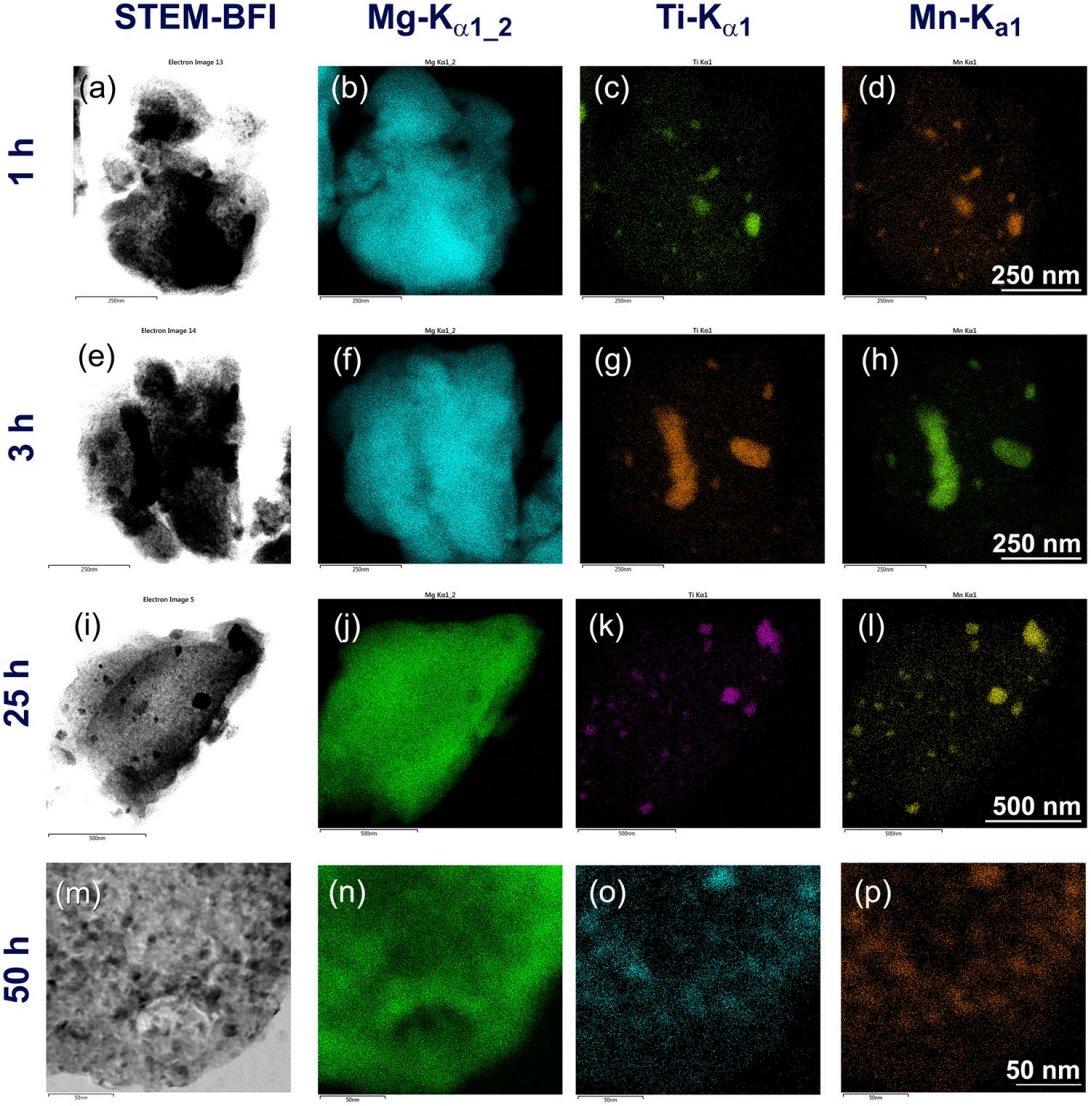
Influence of RBM time on morphological and elemental distributions beyond the nanoscale level for nanocomposite MgH_2_/5 wt.% TiMn_2_ powders. The STEM-BFIs for the powders obtained after 1, 3, 25 and 50 h of RBM time are shown in (**a**,**e**,**i**,**m**), respectively. The Mg-Kα_1-2_, Ti-Kα_1_ and Mn-Kα_1_ for the powders obtained after 1 h and 3 h (early stage of RBM time) are shown together in (**b**–**d**,**f**–**h**), respectively. The EDS elemental mapping images shown in (**j**–**p**) are corresponding to Mg-Kα_1-2_, Ti-Kα_1_ and Mn-Kα_1_ for those samples obtained after 25 h (**i**) and 50 h (**m**), respectively.

In order to understand the distribution effect of TiMn_2_ powders into MgH_2_ matrix on the kinetics behaviors and thermal stability of the hydride phase, carful energy-dispersive X-ray spectroscopy (EDS) elemental mapping experiments were conducted for the powders obtained after the early (1–3 h), intermediate (25 h) and final (50 h) stages of RBM processing time. The composite powders obtained after 1 h of RBM revealed a fine MgH_2_ aggregates (Fig. 2(a) and (b)) doped with thick layer particles of TiMn_2_ (Fig. 2(c) and (d)). After 3 h of RBM (Fig. 2(e)), TiMn_2_ particles (Fig. 2(g) and (h)) were deformed into flake-like morphology and heterogeneously distributed into the MgH_2_ matrix (Fig. 2(f)). The EDS analysis of the powders obtained after the early stage of milling indicated that the concentration of the kinetic-modifier phase (TiMn_2_) was widely varied from particle to particle and even within the particle itself. The local chemical analysis of TiMn_2_ measured with an average area of 0.3 μm was in the range between 0.7 and 64 wt. %.

Increasing the RBM time (25 h) led the large TiMn_2_ flaky particles to be disintegrated into smaller particles, ranging in size between 10 to 150 nm in diameter, as shown in Fig. 2(i–l). However, these metallic particles are heterogeneously embedded into the MgH_2_ matrix (Fig. 2(j)). The TiMn_2_ concentration in the host MgH_2_ matrix was significantly improved, as indicated by the near concertation values varied from 3.6 to 7.8 wt. %. Toward the end of the RBM time, the TiMn_2_ particles (dark lenses presented in Fig. 2(m)) revealed significant changes in their shapes and possessed spherical-like morphology with fair distributions (Fig. 2(o), and (p)) into MgH_2_ matrix (Fig. 2(n)). This morphological improvement of TiMn_2_ particles was followed by a dramatic deduction in their sizes laid within a narrow size distribution range between 5 nm to 27 nm (Fig. 2(m,o,p)). Accordingly, and based on EDS local analysis of different locations in several particles, the concentration of TiMn_2_ in the MgH_2_ matrix was outstandingly improved to be in the range between 4.8 to 5.3 wt. %.

### Thermal stability

**Figure 3:**
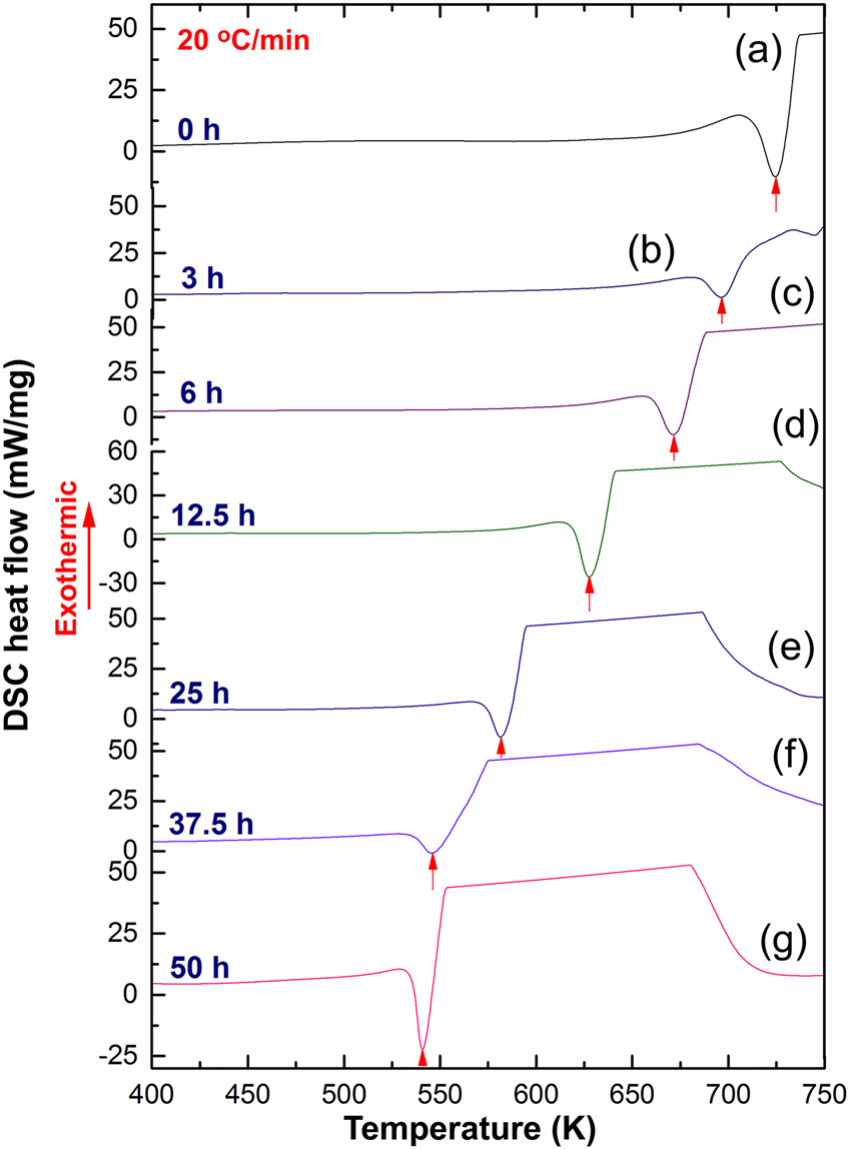
Effect of RBM time on the thermal stability of MgH_2_/5 wt.% TiMn_2_ powders obtained after selected RBM time. DSC curves of MgH_2_ powder obtained after 200 h of RBM time and then mechanically doped with 5 wt.% TiMn_2_ powder for (**a**) 0 h, (**b**) 3 h, (**c**) 6 h, (**d**) 12.5 h, (**e**) 25 h, (**f**) 37.5 h, and (**g**) 50 h.

Differential scanning calorimetry (DSC) performed at a constant heating rate of 20 °C/min under helium gas flow of 100 ml/min was employed to investigate the effect of RBM time and TiMn_2_ additive on the decomposition temperature (dehydrogenation temperature at normal pressure) of as-synthesized MgH_2_ powders. The DSC trace of as-synthesized MgH_2_ powders obtained after 200 h of RBM revealed a broad endothermic event laid at a peak temperature of 728 K, as shown in Fig. 3(a). This endothermic peak notably shifted to the lower temperature side (697 K) upon milling for short time (3 h), as displayed in Fig. 3(b). The peak decomposition temperature for those samples obtained after 6 h (Fig. 3(c)) and 12.5 h (Fig. 3(d)) were 671 K and 628, respectively. The tendency of MgH_2_ powders to be decomposed at lower temperature (581 K) with increasing the RBM time was continuously occurred during the intermediate stage of RBM (25 h), as displayed in Fig. 3(e). During the final stage of RBM (37.5 h) the decomposition temperature was retreated to 546 K, as elucidated in Fig. 3(f). This value did not remarkably changed (541 K) for the nanocomposite powders obtained after 50 h of RBM time (Fig. 3(g).

#### Apparent activation energy of dehydrogenation

**Figure 4:**
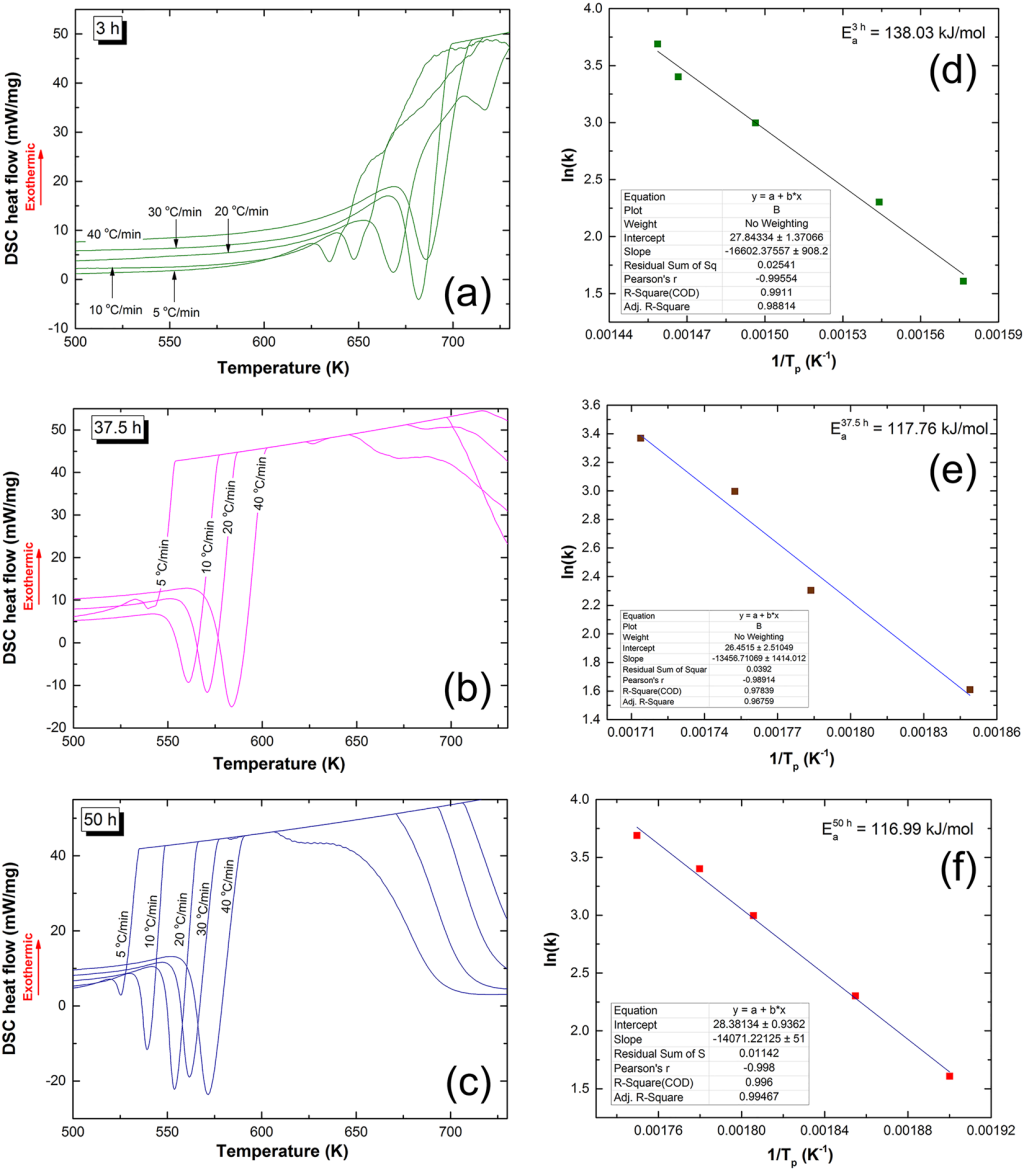
Dependence of activation energy on the RBM time for nanocomposite MgH_2_/5 wt.% TiMn_2_ powders. DSC curves of the nanocomposite powders obtained after RBM time of (**a**) 3 h, (**b**) 37.5, and (**c**) 50 h. The apparent activation energy (E_a_) was calculated from the slope of line shown in the Arrhenius plots, displayed in (**d**), (**e**,**f**) for the samples milled for 3 h, 37.5 h, and 50 h, respectively.

In order to realize the effect of doping MgH_2_ with 5 wt. % TiMn_2_ powders on the apparent activation energy (E_a_) of the metal hydride phase, individual DSC experiments were conducted with different heating rates (5, 10, 20, 30 and 40 °C/min). In this study the effect of RBM on E_a_ were investigated for all samples obtained after different milling stages. Figure 4(a) to (c) displayed selected DSC curves conducted at different heating rated (k) for 3 samples obtained after selected RBM times (3 h, 37.5 h, and 50 h). All the scans revealed single endothermic events related to the decomposition of MgH_2_ into metallic Mg and hydrogen gas, as confirmed by XRD technique. While the peak height increased proportionally with the increasing the k from 5 °C/min to 40 °C/min, the peak temperatures (T_p_) were significantly shifted to the higher temperature side, as presented in Fig. 4(a–c)). The E_a_ of dehydrogenation related to each sample was calculated according to the Arrhenius equation^44^:

1 $${{\rm{E}}}_{{\rm{a}}}=-{\rm{RT}}\,\mathrm{ln}(k/k0)$$

where k is a temperature-dependent reaction rate constant, R is the gas constant, and T is the absolute temperature. The E_a_ values were determined by measuring the T_p_ corresponded to the different k and then plotting ln(k) versus 1/T_p_. The E_a_ values were then obtained from the slope of line (−E/R, where R is the gas constant).

Based on these measurements, the nanocomposite powders obtained after 3 h of RBM time showed a high E_a_ value (138.03 kJ/mol), as shown in Fig. 4(d). This indicates a high thermal stability of the powders against decomposition. In contrast, E_a_ of nanocomposite MgH_2_/5 wt.% TiMn_2_ powders obtained after 37.5 h (Fig. 4(e)) of RBM showing a lower value (117.76 kJ/mol), indicating a significant destabilization of the MgH_2_ upon high-energy ball milling with TiMn_2_ phase. The E_a_ value did not show notable improvement (116.99 kJ/mol) upon increasing the RBM time to 50 h, as elucidated in Fig. 4(f). The apparent E_a_ of our system is closed to the reported one for MgH_2_/5 wt. % Zr_70_Ni_30_Pd_10_ powders (92 kJ/mol)^4^. However, our system showed lower E_a_ value when compared with pure MgH_2_^45^ (164 kJ/mol)^40^, Mg_85_In_5_Al_5_Ti_5_ (125.2 kJ/mol)^46^, and Mg_17_Ba_2_ (173.92 kJ/mol)^47^, systems, it is well above than those corresponding values reported for MgH_2_ powders coated by Ti-based thin films (30.8 kJ/mol)^48^, CeH_2.73_-MgH_2_-Ni nanocomposite (63 kJ/mol)^49^, Mg- TiVMn (85.2 kJ/mol)^50^, Mg- Li_2_TiO_3_ (84 kJ/mol)^51^, and MgH_2_-Ta_2_O_5_ (74 kJ/mol)^52^ systems.

**Figure 5:**
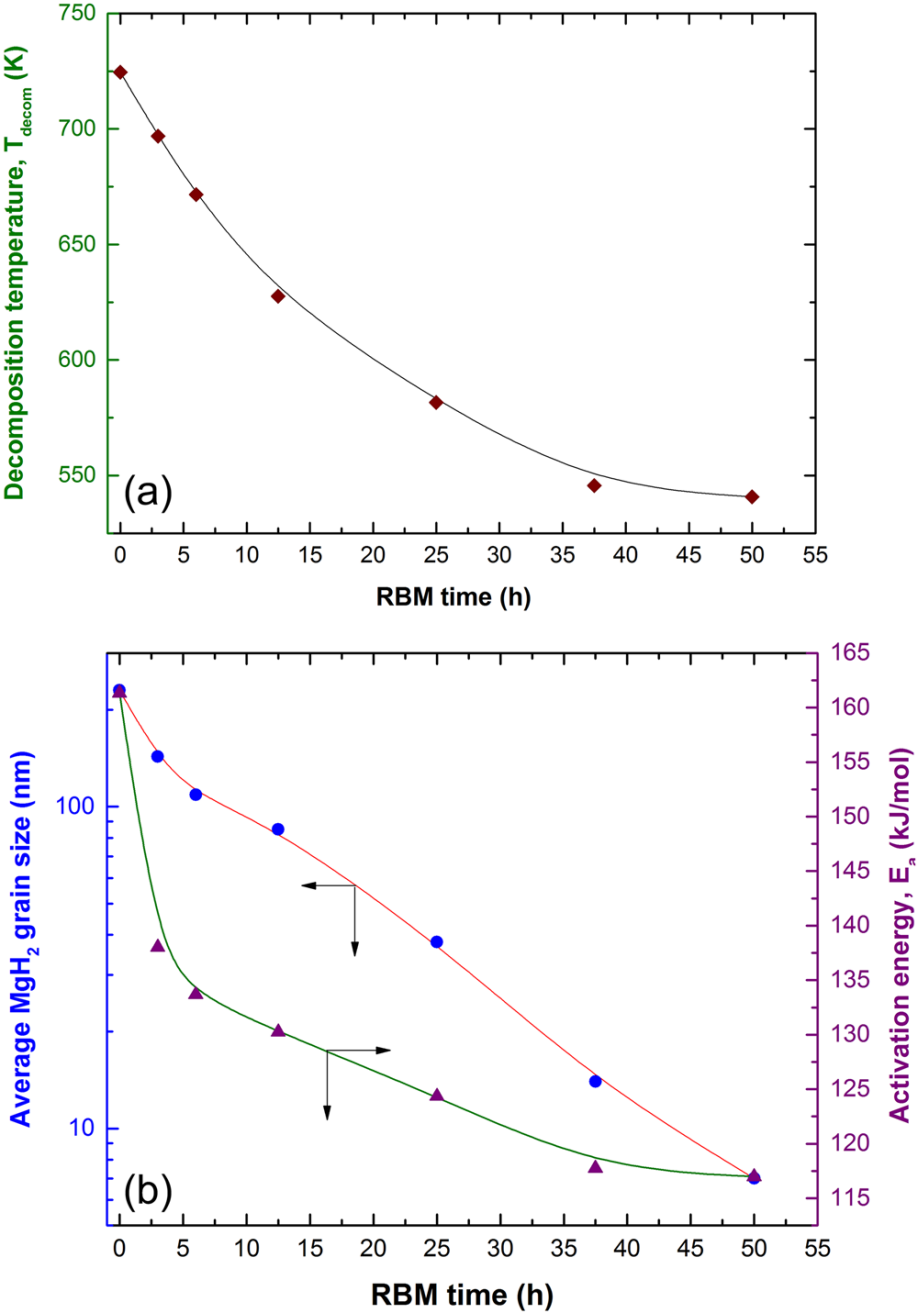
Correlation between RBM time, decomposition temperature, MgH_2_ grain size and activation energy of nanocomposite MgH_2_/5 wt.% TiMn_2_ powders. (**a**) Influence of RBM time on the decomposition temperature, T_decom_ for nanocomposite MgH_2_/5 wt.% TiMn_2_ powders. The effect of RBM time on MgH2 grain size and activation energy, E_a_ of nanocomposite MgH_2_/5 wt.% TiMn_2_ powders are elucidated in (**b**).

Figure 5 summarizes the DSC experiments by presenting the effect of RBM time on T_p_ (Fig. 5(a)). It is well established that high-energy ball milling process is a typical destabilization process^11,17,18,53^ generating several lattice imperfections (e.g. dislocations, staking faults, point defects, etc.) in the milled powders^10^. In thermodynamics point of view, the existence of these defects are favorable for destabilize the stable MgH_2_ phase into a metastable phase^45–47^, where the hydrogen gas can be released at lower temperature values. As the milling time increases from 0 h to 12.5 h (early stage of RBM), the grain size of MgH_2_ was drastically decreased from 231 nm to 84 nm, as displayed in Fig. 5(b). The T_p_ was not affected by such deduction on the MgH_2_ grain size only, but in fact it was greatly affected by the continuous morphological improvement taken place with increasing RBM time, as discussed in Fig. 2. The morphological effect including grain size refining on improving the hydrogenation/dehydrogenation kinetics of MgH_2_ binary system was demonstrated by many authors^48–51^. During this early stage of RBM time, the T_p_ was dropped from 725 K to 628 K, as shown in Fig. 5(a). Moreover, the continuous size reduction happened in this early stage of milling led to a notable improvement in E_a_ that tended to decrease from 162 (0 h) to 130 kJ/mol (12.5 h), as elucidated in Fig. 5(b). The influence of grain size reduction on E_a_ and kinetics is well established and attributed to the short-diffusion bathes of hydrogen atoms^27^. We should emphases that intermetallic TiMn_2_ hard particles did not only played a heterogeneous catalytic role for improving the poor kinetics of MgH_2_, but also considered as micro-milling media leading to accelerate the grain size refinement of the metal hydride powders.

During the intermediate stage of RBM (25 h) the MgH_2_ grain size was reduced to 38 nm (Fig. 5(b)), where T_p_ and E_a_ reached to lower values of 582 K (Fig. 5(a)) and 124 kJ/mol (Fig. 5(b)), respectively. Toward the end of the RBM processing time (37.5 h–50 h) the MgH_2_ grain continued their tendency to be reduced in sizes (14 nm–7 nm) according to the double successive effects of balls- and TiMn_2_-micro milling media. Further destabilization of MgH_2_ taken place during this final stage of RBM, referred by the reduction occurred in T_p_ values (546 K–540 K), as shown in Fig. 5(a). Most importantly and as a result of MgH_2_ grain refinement, Ea tended to drop into a lower value (116–117 kJ/mol), as elucidated in Fig. 5(b).

### Hydrogenation/dehydrogenation kinetics behavior

The improvement of kinetics related to hydrogen absorption and desorption processes attained upon mechanical doping MgH_2_ with 5 wt. % TiMn_2_ powders were monitored after different RBM time, using Sievert’s method^45^.

#### Kinetics of hydrogenation

**Figure 6:**
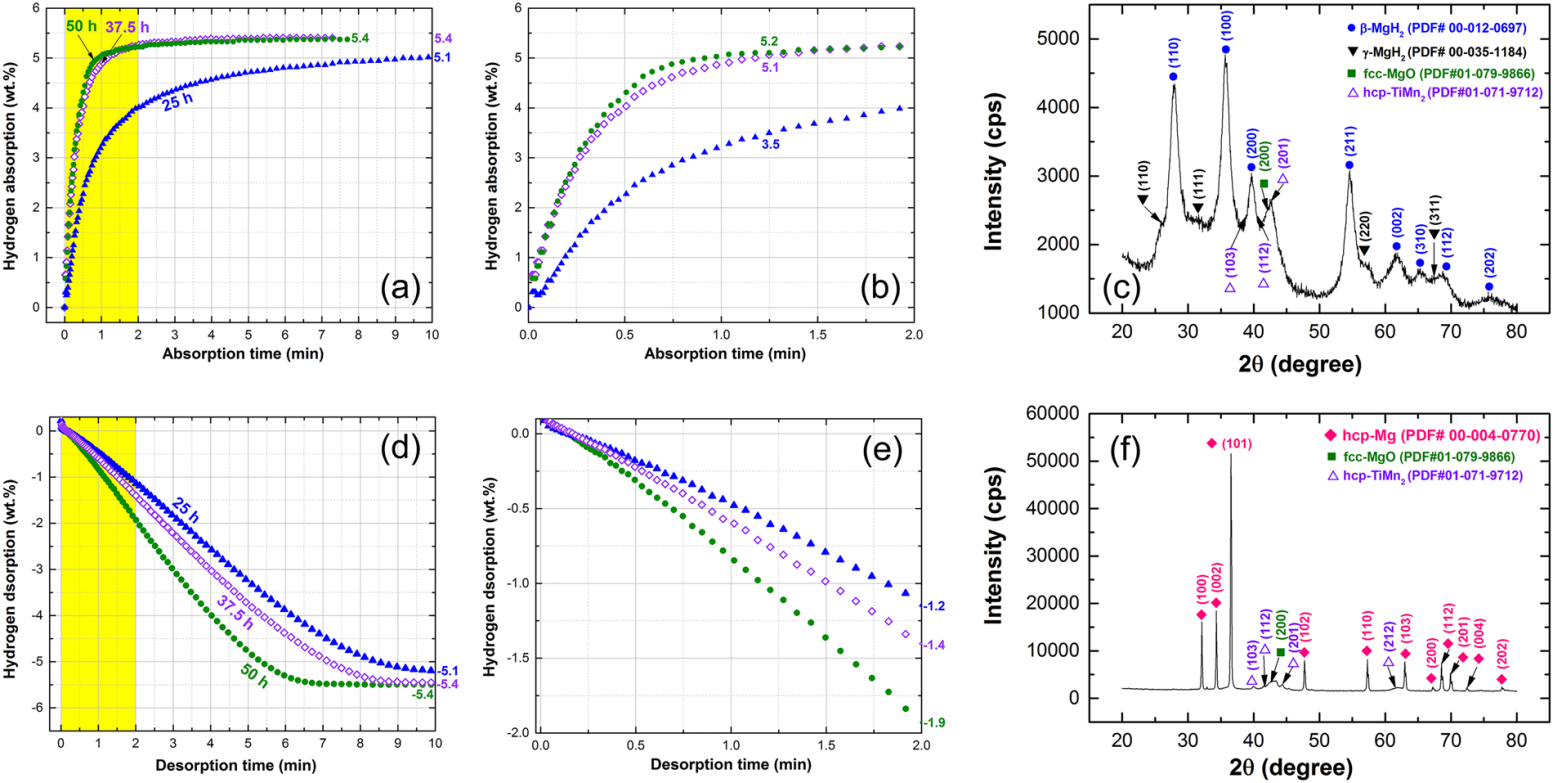
Effect of RBM time on the hydrogenation/dehydrogenation Kinetics measured at 275 °C for nanocomposite MgH_2_/5 wt.% TiMn_2_ powders. The full-time-scale (10 min) of hydrogenation and dehydrogenation kinetics examined at 275 °C are displayed in (**a**,**d**), respectively. The first-2 min corresponding to the yellow shaded zones shown in (**a**,**d**) are displayed in (**b**,**e**), respectively. XRD patterns of the nanocomposite samples obtained after 50 h and then subjected to hydrogenation, and dehydrogenation processes at 275 °C are presented in (**c**,**f**), respectively.

The hydrogenation kinetics behavior investigated at 250 °C under 10 bar H_2_ gas pressure for nanocomposite MgH_2_/5 wt.% TiMn_2_ powders obtained after selected RBM time are shown in Fig. 6(a). The shaded zone indexed in Fig. 6(a) is elucidated in Fig. 6(b) with a different time scale. After 25 h of RBM, the sample absorbed 3.5 and 4 wt. % H_2_ after 1.25 and 4 min, respectively as shown in Fig. 6(b). As previously discussed in Fig. 5(b), increasing the RBM time resulting a significant decreasing in the MgH_2_ grain size, allowing an easier hydrogenation process. Accordingly, the sample obtained after 37.5 h of RBM time showed a significant development on absorbing 5.1 wt. % H_2_ after only 1.25 min (Fig. 6(b)). Marginal improvement (5.4 wt. % H_2_) was notified with increasing the absorption time to 3 min (Fig. 6(a)). Further increasing of the absorption time (~7.75 min) did not cause any improvement on hydrogen uptake and the sample saturated at 5.4 wt. % H_2_, as elucidated in Fig. 6(a). However, longer RBM time (50 h) did not show a major beneficial effect on the hydrogen uptake (5.4 wt. % H_2_) and hydrogenation kinetics, the sample possessed a notable ability to absorb higher H_2_ value with shorter time (~4.7 wt. %/0.6 min), as displayed in Fig. 6(b). This value is higher than the one shown by the sample obtained after 37.5 h of RBM (4.4 wt. % H_2_) at the same absorption time. It should be mentioned that Mg/5 wt.% Ta_2_O_5_ binary system can uptake about 6 wt.% H_2_ within 60 min at 100 °C^52^. Doping MgH_2_ with 5 wt.% Li_2_TiO_3_ led to enhance the hydrogenation kinetics conducted at 300 °C to absorb 5.5 wt. % H_2_ within 5 min^51^. For Mg doped with 30 wt.% TiMn_1.5_, 2 min was required to perform complete hydrogenation (~4.4 wt. % H_2_) at 300 °C^54^. MgH_2_-VTiCr and MgH_2_-TiMn_2_ systems possessed nearly complete absorption of ~5.5 and 4.5 wt. % H_2_, respectively within 15 min at 300 °C^52^. It has been reported that doping MgH_2_ with 5 wt.% TiMn_2_ led to conduct hydrogenation of ~4.8 wt. % H_2_ within 250 min at 100 °C^55^. Doping MgH_2_ with 20 wt % Ti_0.4_Cr_0.15_Mn_0.15_V_0.3_ led to absorb 5.7 wt.% H_2_ in 100 min at low temperature (100 °C)^56^.

The XRD pattern of 50 h sample taken after hydrogenation process at 250 °C is shown in Fig. 6(c). The sample revealed a domain structure of γ-MgH_2_ coexisted with small molecular fractions of γ-MgH_2_. Moreover, the XRD analysis did not confirm the formation of any intermediate or reacted phases (MgTi, TiH_2_, MgTiH) where the TiMn_2_ additive powder maintained its original hcp-structure (PDF file #01-071-9712), as displayed in Fig. 6(c). This implies that TiMn_2_ additive can be considered as heterogeneous catalysts, in which it enhanced the hydrogenation/dehydrogenation kinetics of MgH_2_ without entering into a chemical reaction with MgH_2_ matrix.

#### Kinetics of dehydrogenation

In order to get the complete picture of TiMn_2_ effect on enhancing the behavior of MgH_2_ powders, The kinetic of hydrogen releasing for the samples obtained after 25 h, 37.5 h, and 50 h of RBM time were carefully investigated at 250 °C at 200 mbar H_2_ gas pressure. Figure 6(d) and (e) present the effect of RBM time on the desorption kinetics of nanocomposite MgH_2_/5 wt. % TiMn_2_ powders. After very short desorption time (1 min) the 3 selected samples showed poor dehydrogenation kinetics, indicated by the low values of H_2_ released (−0.4 to 0.8 wt. %), as displayed in Fig. 6(e). Significant improving on the hydrogenation kinetics was attained for 50 h sample that desorbed −1.9 wt. % H_2_ within 2 min (Fig. 6(e)). In contrast, a moderate improvement could be notified for the sample obtained after 37.5 h and 25 h that succeed within 2 min to discharge −1.4 and −1.2 wt. % H_2_, as presented in Fig. 6(e). Increasing the absorption time led to enhance the ability of the 3 samples for desorb more H_2_, exemplified by the H_2_ wt. % absorbed at 3 min for 25 h (−1.8), 37.5 h (−2.2), and 50 h (−3), as shown in Fig. 6(d). After 7 min, the 50 h sample attained a saturated value of hydrogen released (−5.4 wt. %), where 25 h and 37.5 h samples released only −4.5 and 4.9 wt. % H_2_, respectively (Fig. 6(d)). Where there was no remarkable change in the desorbed hydrogen could be notified for 50 h sample upon increasing the desorption time between 7 to 10 min, the 25 h and 37.5 h samples tended to getting changes in the H_2_ desorbed values that reached to −5.1 and −5.4 wt.% after 10 min of the desorption time (Fig. 6(d)). Previously published MgH_2_/5 wt.% TiMn_2_ system possessed fast dehydrogenation ability at 300 °C to discharge 4.5 wt. % H_2_ within only 2 min^55^. Different research group reported that MgH_2_/5 wt.% TiMn_2_ system can desorb its storage capacity of 4.8 wt.% H_2_ in 6 min at 270 °C^45^. It was demonstrated that MgH_2_/30 wt.% TiMn_1.5_ system can achieve complete absorption/desorption processes in 20 min at 250–300 °C with rather poor hydrogen storage capacity (4.3 wt. % H_2_)^54^. MgH_2_/Ti_0.4_Cr_0.15_Mn_0.15_V_0.3_ composite system was able to discharge its storage capacity (5.7 wt. % H_2_) within 30 min at 100 °C^56^.

The crystal structure related to 50 h sample obtained after the dehydrogenation test was examined by XRD. The sample revealed sharp Bragg peaks corresponding to hcp-Mg (PDF file #00-004-0770) coexisted with fine hcp-TiMn_2_ (PDF file #01-071-9712) particles having broad Bragg peaks patterns, as shown in Fig. 6(f). Neither reacted nor intermediate phases could be detected, implying the absent of any undesirable reactions during the dehydrogenation process.

### Cyclability of hydrogen absorption/desorption

**Figure 7:**
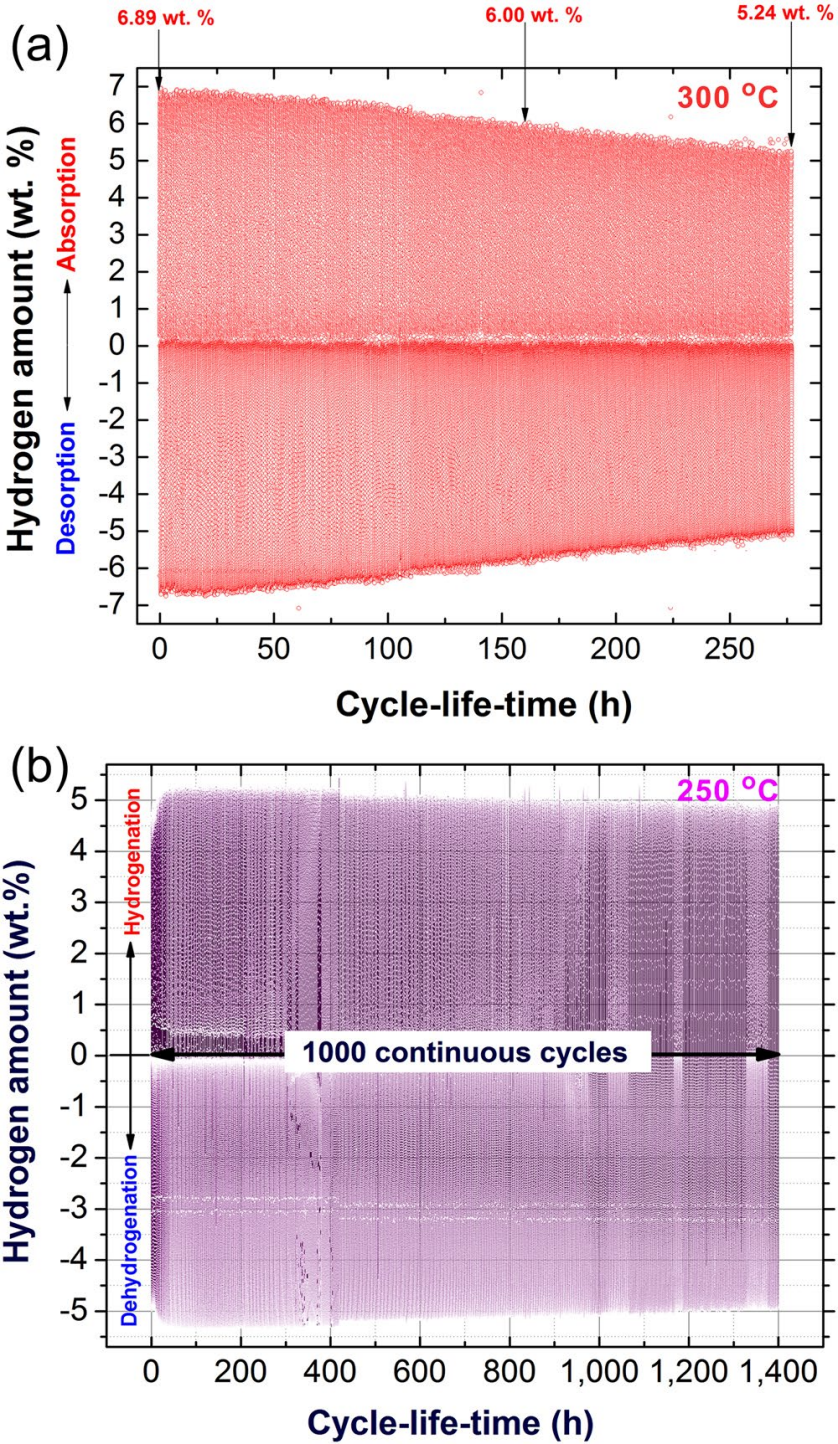
Effect of TiMn_2_ additive on the cyclability of MgH_2_ powders. Cycle-life-time conducted at 300 °C for (**a**) nanocrystalline MgH_2_ powders, and at 250 °C for (**b**) nanocomposite MgH_2_/5 wt.% TiMn_2_ powders.

Measuring the cycle-life-time, which reflects the capability and performance of synthesized nanocomposite MgH_2_/5 wt.% TiMn_2_ powders for achieving continuous hydrogenation/dehydrogenation cycles was investigated. For comparison, the cycle-life-time test of pure MgH_2_ powders obtained after 200 h of RBM time (before doping with TiMn_2_ powders) was conducted and the results are displayed in Fig. 7(a). To ensure the cyclic continuity and to improve the of hydrogenation/dehydrogenation kinetics of pure MgH_2_, the test was achieved at high temperature (300 °C). The pressures used for hydrogenation/dehydrogenation processes were 8/0.2 bar, respectively.

**Figure 8:**
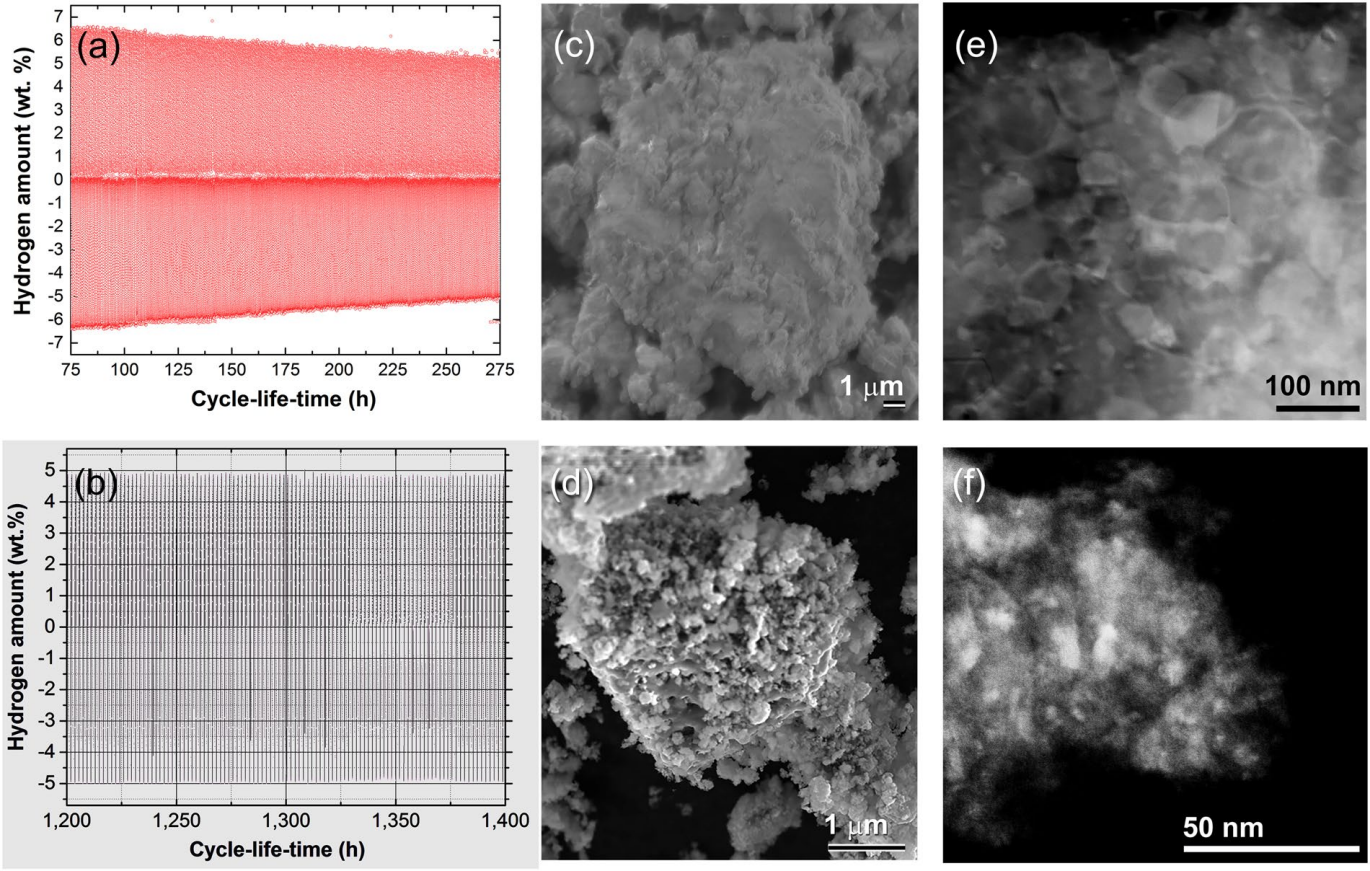
Influence of cycle-life-time and TiMn_2_ additives on the morphological characteristics of the powders. The cycle-life-time of the last 200 h for (**a**) nanocrystalline MgH_2_ powders, and (**b**) nanocomposite MgH_2_/5 wt.% TiMn_2_ powders. The FE-SEM micrographs of the powders obtained after completion the cyclic hydrogenation tests displayed in (**a**,**b**) are shown in (**c**,**d**), respectively. STEM-DFIs of the powders obtained after completion 275 h cycle-life-time (MgH_2_ powders) and 1400 h (MgH_2_/5 wt.% TiMn_2_ powders) are shown in (**e**,**f**), respectively.

However, the as-prepared nanocrystalline MgH_2_ powders possessed excellent hydrogen storage capacity of about 6.9 wt. % (Fig. 7(a)), the powders failed to maintain their capacity. This is indicated by a monotonic degradation on the storage capacity upon increasing the cycle-life-time, reaching a lower value (6.3 wt. % H_2_) after only 75 h, as shown in Figs 7(a) and 8(a). When the powders were subjected to longer cycle-life-time in the range between 75 −275 h (Figs 7(a) and 8(a)), the hydrogen storage capacity was severely degregated to reach a lower value of 5.4 wt. % H_2_, after 200 h (Fig. 8(a)). Toward the end of the test (275 h), the MgH_2_ powders were disable to store more than 5.24 wt. % H_2_, as elucidated in Fig. 7(a). The tendency of nanocomposite MgH_2_/5 wt.% TiMn_2_ powders to manifest hydrogen storage degradation was almost absent even after 1400 h of continuous hydrogen uptake/discharge, as presented in Figs 7(a) and 8(b).

In order to realize the reasons responsible for such severe degradation, the morphology of the material powders obtained after 275 h of cycle-life-time (Mg powders) were examined by FE-SEM technique. Noticeably, the metallic powders after this test tended to agglomerate and composite larger particles with average size of 13 μm in diameter, as presented in Fig. 8(c). This agglomeration behavior was a result of the continuous applications of rather high hydrogen pressure on the powders at a high temperature (300 °C). Moreover, the topology of the powder was solid, where the pores facilitating the hydrogen diffusion into/out the powder were absent (Fig. 8(c)). The absent of favorable pores and cavities degrade the performance of MgH_2_ powders to maintain their hydrogen storage capacity. Unluckily, and according to the application of high temperature and pleasure the metallic Mg grains revealed severe grain growth with an average diameter of 80 nm, as shown in the STEM-dark field image (DFI) in Fig. 8(e).

The beneficial effect of doping nanocrystalline MgH_2_ with 5 wt. % TiMn_2_ powders on the hydrogenation/dehydrogenation cyclability conducted at 250 °C under hydrogenation/dehydrogenation pressure of 8/0.2 bar is clearly realized in Fig. 7(b). The nanocomposite powders possessed very high performance for achieving 1400 cycle-life-time (~1000 continuous cycles) without severe degradation, as displayed in Fig. 7(b). During the first 400 h, the sample maintained its original hydrogen storage capacity (5.4 wt.%), that was slightly decreased to about 5 wt. % H_2_ after 900 h, as shown in Fig. 7(b). Further unserious decreasing can be seen after 1200 h when the hydrogen storage capacity dropped a little to the level of 4.95 wt. % H_2_, as presented in Fig. 7(b). The last 200 h of the cycle-life-time test is individually displayed in Fig. 8(b). During this last stage of the test, the nanocomposite powders maintained their hydrogen storage capacity at almost a constant value of 4.95 wt. %, as elucidated in Fig. 8(b). Moreover, the kinetics of hydrogenation/dehydrogenation processes remaining constant with no obvious failure or decay.

Since milling MgH_2_ with TiMn_2_ powders did not lead to the formation of any reacted intermediate phase (Fig. 6(c) and (f)) responsible for enhancing the kinetics and performance of charging/discharging hydrogen cyclability, the justification beyond such obvious improvements can be related to a morphological reason. Figure 8(d) presents the FE-SEM of nanocomposite MgH_2_/5 wt.% TiMn_2_ powders after completion of 1000 hydrogenation/dehydrogenation cycles. The aggregated powders consists of ultrafine MgH_2_/TiMn_2_ particles, ranging in sizes between 0.22 μm to less than 0.5 μm in diameter, as shown in Fig. 8(d). In addition, TiMn_2_ fine particles (~5 nm in diameter) maintained their tendency for adhering onto the MgH_2_ surface of powders, even after 1400 h of cycle-life-time, as shown in the STEM-DFI presented in Fig. 8(f). Comparing the STEM-DFI presented in Fig. 8(d) with that one for nanocomposite powders before conducting the cycle life-time test (Fig. 2(m–p)) led us to confirm the absence of any undesired grain growth for both MgH_2_ grain matrix and TiMn_2_ particles. The limited grain growth seen in MgH_2_ grains is probably attributed to the distribution of TiMn_2_ particles (grain-growth inhibitors) into the MgH_2_ matrix. It was pointed out by Yao *et al*.^57^ that hydrogen diffusion is much faster when Mg grains were in the nanoscale level.

Based on these results, it can be concluded that one advantage of using hard intermetallic compounds such as TiMn_2_, refractory metal carbides and amorphous alloys for improving the kinetics behavior and cycle-life-time of MgH_2_ powders is related to the tendency of these hard particles to surround MgH_2_ grains leading to block their attitude of growing at high temperature and pressure. Another advantage of TiMn_2_ intermetallic compound additives with their nano-dimensional particle size is the improvement of the cyclability performance for MgH_2_ system. This present system can be considered as one of the most reliable performance system among the most well known system, exemplified by MgH_2_-5 wt. % of Ti_0.4_Cr_0.15_Mn_0.15_V_0.3_ (73 cycles, 290 °C)^56^, TiMn_2_ (100 cycles, 300 °C)^55^, VTiCr (100 cycles, 300 °C)^55^, FeTi (500 cycles, 200 °C)^58^, metallic glassy of Zr_70_Ni_20_Pd_10_ (100 cycles, 200 °C)^4^, 10 wt.% of big-cube Zr_2_Ni (2546 cycles, 250 °C)^21^, ZrNi_5_ (600 cycles, 275 °C)^45^, 5Ni/5Nb_2_O_5_ (180 cycles, 250 °C)^59^, and 5TiC/5Fe-12Cr (530 cycles, 275 °C)^25^.

### Integrated hydrogen storage system for fuel cell Applications

Our present work have two objectives; the first is focused on synthesizing and characterizing a nanocomposite MgH_2_/5 wt. % TiMn_2_ system, where the second objective is focused on utilizing the as-synthesized nanocomposite powders manufacturing of a complete hydrogen storage system for fuel cell applications. To attain this purpose, a simple hydrogen storage tank was manufactured (Supplementary Materials, Fig. S1) composited of high-pressure hollow vessel made of pure titanium (Ti) metal where a hollow–graphite mould with an inner diameter of 10 mm was inserted into the vial. The powders were then charged into hollow-graphite mould and the system was sealed with copper metal gasket inside the glove box. A high pressure ball valve was perfectly installed into the tank cap’s to allow hydrogen gas releasing and charging.

**Figure 9:**
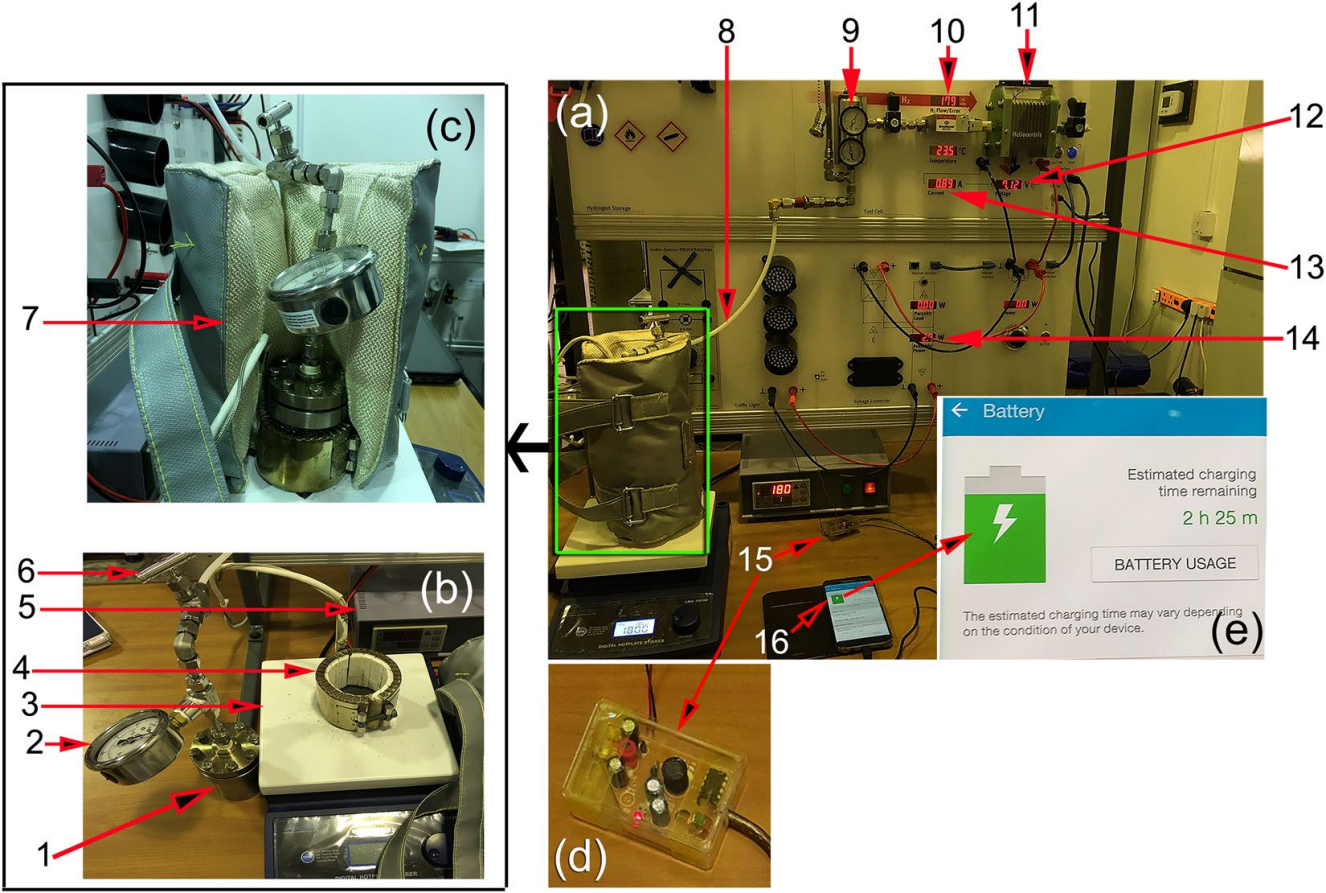
Integrated hydrogen storage tank invented in the present work coupled with 40 W/4.5 A proton exchange membrane (PEM) fuel cell. (**a**) prototype bench-lab of hydrogen storage tank (**b**,**c**) designed and manufactured in the present work integrated with PEM fuel cell. The system consists (1) hydrogen storage tank filled with nanocomposite MgH_2_/5 wt.% TiMn_2_ powders, (2) high-pressure hydrogen-gauge, (3) hot-plate, (4) jacket heater with copper heating-elements, (5) jacket heater temperature controller, (6) hydrogen uptake/release controlling valve (7) jacket temperature insulator, (8) tube connecting the hydrogen storage system with the fuel-cell unit, (9) H_2_-PEM input gauge monitor, (10) hydrogen-flow indicator, (11) 40 W/4.5 A PEM-type fuel cell, (12) voltage indicator, (13) electric current indicator, (14) available power produced by the fuel cell, (15) 5 V voltage regulator (**d**), (16) cell-phone device, showing cell phone battery charging monitor with a different magnification in (**e**).

Figure 9 presents photographs related to our experimental set up of the integrated hydrogen storage system including the Ti-vial placed into a gas-temperature control heater (Fig. 9(a) and (b)) with a jacket temperature insulator (Fig. 9(c)). The tank was then mounted on a temperature controlled hotplate (Fig. 9(c)). The hydrogen storage tank was connected 40 W/4.5 A proton exchange membrane (PEM) fuel cell (Fig. 9(a)) through a pipeline allowing the released hydrogen gas form the tank to be passed to the PEM-fuel cell, as shown in Fig. 9(a). The converted electrical energy required to charge the battery of a cell phone device through 5 V voltage regulator (Fig. 9(a)). The PEM-fuel cell system is controlled and operated with a software where the data output corresponding to hydrogen flow rate, voltage and current were obtained and stored.

**Figure 10:**
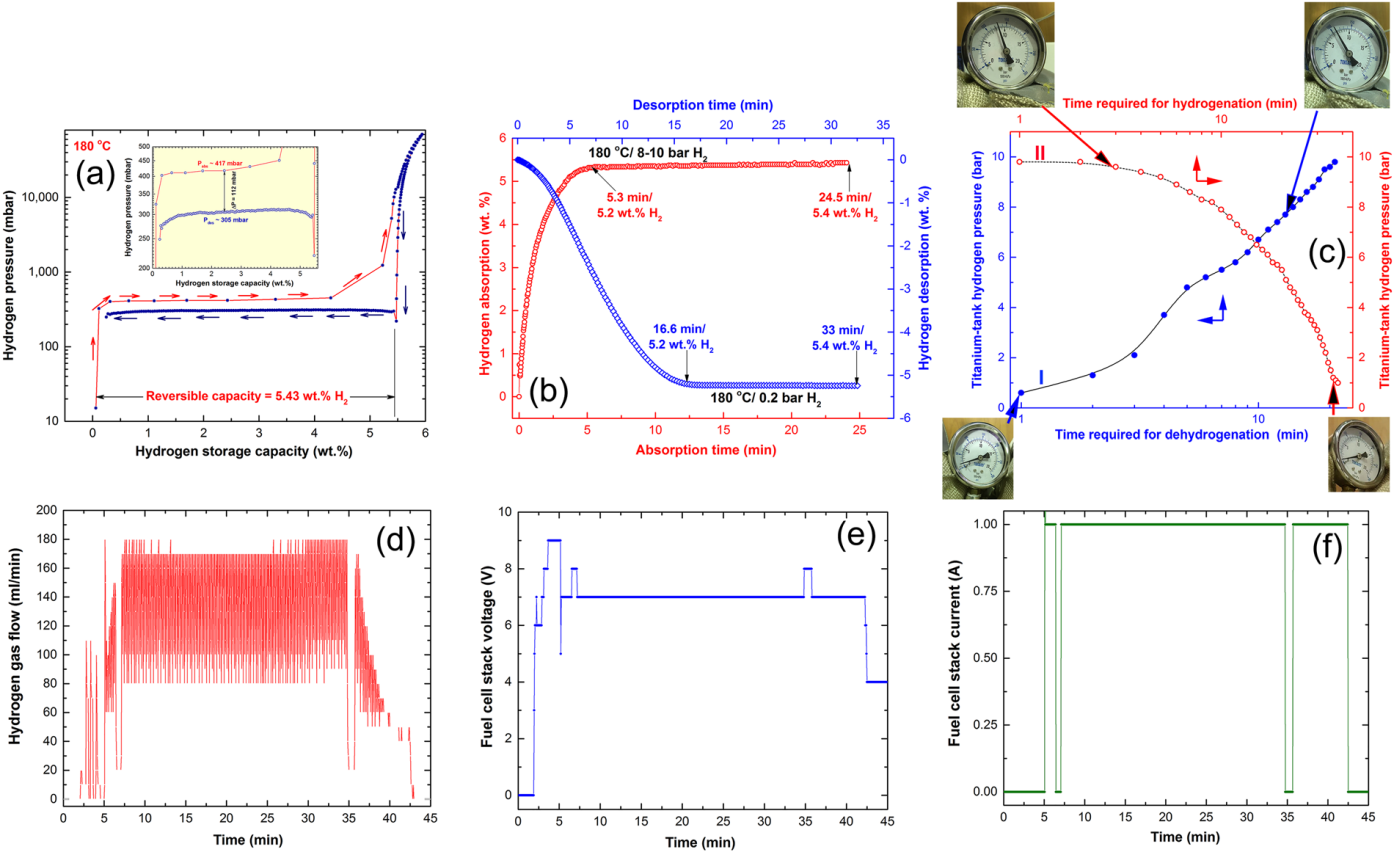
Characterization of fuel cell system fed by hydrogen gas flow released from nanocomposite MgH_2_/5 wt.% TiMn_2_ powders. PCT and hydrogenation/dehydrogenation kinetics curves conducted at 180 °C are shown in (**a**,**b**), respectively. The time required for hydrogenation/dehydrogenation conducted at 180 °C for Ti-tank is plotted vs the corresponding pressure in (**c**). The hydrogen gas flow provided during charging cell-phone’s battery is presented in (**d**). Correlation between charging time and fuel cell- stack (**e**) current, and (**c**) voltage.

Before starting the fuel-cell testing, the powders were obeyed to pressure-composition-temperature (PCT) analysis ensure the possibility of dehydrogenation process at a low temperature (180 °C) and to investigate the pressure required to achieve the decomposition process, MgH_2_/5TiMn_2_ powders obtained after 50 h of milling. In the PCT experiment, the sample was firstly activated at high temperature (350 °C) that corresponding to about 35 bar of H_2_ for 12 h. The powders were then charged with hydrogen at 180 °C, where the corresponding pressure reached to 0.4 bar, as shown in Fig. 10(a). Under this low temperature (180 °C) the fully charged powders tended to discharge their hydrogen storage capacity (5.43 wt.%) at 0.3 bar, as displayed in Fig. 10a. The PCT experiments was repeated 5 times under the same conditions to ensure the reprodcutability of the results. It is worth to be mentioned that the presence of minimal pressure-hysteresis gap between the pressure of absorption and desorption (~0.1 bar) indicates the powders’ ability to achieve long term of cycle-life-time without failure, as was previously presented (Fig. 7b). Moreover, and in part to ensure our results, new set of kinetic measurements were conducted at 180 °C for both hydrogenation and dehydrogenation reactions taking place under 8–10 bar/0.2 bar (Fig. 10(b)). The possibility of achieving both reactions at 180 °C was approved, however, both reactions showed rather slow kinetics when compared with the results obtained at 250 °C (Fig. 6(a) and (d)).

Prior to fuel cell experiments processing start, the nanocomposite MgH_2_/5 wt.% TiMn_2_ powders were activated at 350 °C by charging/discharging hydrogen gas atmosphere at 30 bar/1 bar, respectively for 10 continuous cycles to obtain MgO-surface free nanocomposite powders. A continuous hydrogen gas flow pressurized at 10 bar was introduced at 180 °C to the Ti-tank containing the powders. The Ti-tank was then kept under this temperature (180 °C), where the readings shown by the hydrogen pressure gauge connected to the tank (Fig. 10(c)) was monitored and recorded every 1 min. These readings were used to construct a relationship between the time required for the powders placed to be charged with hydrogen, as shown by the open red symbols presented in Fig. 10(c). Completion of the absorption process was realized when the hydrogen was completely absorbed by the powders and the tank’s pressure dropped to the atmospheric level (Fig. 10(c)). The hydrogen gas cylinder was then removed and disconnected from the tank, where the tank was kept under this 180 °C for almost 40 min. In order to construct a relation between the time required to release hydrogen gas stored in the powder in Ti tank, the readings of the pressure gauge was recorded every 1 min. The monotonical increase of hydrogen pressure inside the closed Ti-tank implies a gradual hydrogen release from the powders, as elucidated by the closed blue symbols shown in Fig. 10(c). The time required to release hydrogen from MgH_2_ powders at 180 °C was about 25 min, as shown in Fig. 10(c). At this temperature, we started to open the valve of the tank shown connected to the PEM-FC system (Fig. 9(a)) with nearly constant hydrogen flow of 175 ml/min (Fig. 10(d)).

Figure 10(d) displays the hydrogen gas flow transported into the PEM-fuel cell. The hydrogen released from the nanocomposite powders was used to feed the PEM-fuel cell through a pipeline, as shown in Fig. 9(a). During the battery charging time (28 min) the hydrogen gas released from the hydrogen storage tank at 180 °C passed delivered into the PEM-fuel cell at constant rate of 175 ml/min, as shown in Fig. 10(d). Providing the fuel cell with such a constant rate of hydrogen gas flow leads to generate constant values of voltage (7 V) and current (1 A), as displayed in Fig. 10(e) and (f), respectively.

In summary, we have synthesized nanocomposite MgH2/5 wt. % TiMn_2_ system with advanced storage capacity (~5.3 wt. % H_2_), hydrogenation/dehydrogenation kinetics and long hydrogen charging/discharging cyclability shown at rather low temperature (250 °C). The new nanocomposite powders were charged into a self-made Ti-tank and employed as a source of hydrogen required to charge a battery of a cell-phone device through a commercial proton exchange membrane (PEM) fuel cell.

## Methods

### Preparation of MgH_2_ powders

Elemental Mg metal powders (~80 μm, 99.8% provided by Alfa Aesar - USA), and hydrogen gas (99.999%) were used as starting materials. An amount of 5 g Mg was balanced inside a He gas atmosphere (99.99%) - glove box (UNILAB Pro Glove Box Workstation, mBRAUN, Germany) and sealed together with fifty FeCr balls into a hardened steel vial (150 ml in volume), using a gas-temperature-monitoring system (GST; supplied by evico magnetic, Germany). The ball-to-powder weight ratio was maintained at 40:1. The vial was then evacuated to the level of 10^−3^ bar before introducing H_2_ gas to fill the vial with a pressure of 50 bar. The reactive ball milling (RBM) process was carried out at room temperature, using a high-energy ball mill (Planetary Ball Mill PM 400 provided by Retsch, Germany). The RBM process was interrupted after selected milling time (3, 6, 12.5, 25, 50, 100, and 200 h) where the vial was opened inside the glove box to take a small amount (~300 mg) of the milled powders for different analysis. Then, the RBM process was resumed, using the same operational conditions shown above.

The as-synthesized MgH_2_ powders were then mixed in the glove with the 5 wt. % of TiMn_2_ shots, using an agate mortar and pestle. Five gram of the mixed powders for each composite system were charged together with fifty Cr-steel balls into the hardened steel vial and sealed under He gas atmosphere. The vial was then filled with 50 bar of hydrogen gas atmosphere and mounted on the high-energy ball mill. The milling process was interrupted after selected time (3, 6, 12.5, 25, 37.5 and 50 h) and the powders obtained after an individual milling time were completely discharged into 8 Pyrex vails for different analysis. Then, new MgH_2_/5 wt% TiMn_2_ mixed powders were charged again and ball milled under the same milling conditions. The contamination contents of Fe and Cr of the powders obtained after 50 h of ball milling were 1.19 and 0.32 wt. %, respectively.

### Powder characterizations

XRD AND HRTEM. The crystal structure of all samples was investigated by XRD with CuKα radiation, using 9 kW Intelligent X-ray diffraction system, provided by SmartLab-Rigaku, Japan. The local structure of the synthesized material powders was studied by 200 kV-field emission high resolution transmission electron microscopy/scanning transmission electron microscopy (HRTEM/STEM, supplied by JEOL-2100F, Japan), which is equipped with Energy-dispersive X-ray spectroscopy (EDS) supplied by Oxford Instruments, UK. In addition to the elemental analysis achieved by EDS approach, we employed ICP technique to get the elemental analysis by a chemical analytical approach.

Thermal stability. Shimadzu Thermal Analysis System/TA-60WS-Japan, using differential scanning calorimeter (DSC) was employed to investigate the decomposition temperatures of MgH_2_ powders with a heating rate of 20 °C/min. The activation energy for of the powders obtained after different RBM time were investigated, using Arrhenius approach with different heating rates (5, 10, 20, 30, 40 °C/min).

#### The hydrogenation/dehydrogenation behaviors

The hydrogen absorption/desorption kinetics were investigated via Sievert’s method^60–62^, using PCTPro-2000, provided by Setaram Instrumentation, France, under hydrogen gas pressure in the range between 200 mbar (for dehydrogenation) to 10 bar (for hydrogenation). The samples were examined at different temperatures of 50, 100, 250, and 275 °C. In the PCT measurements, the dosed pressure in absorption/desorption was gradually increased/decreased by 1000 mbar until equilibrium pressure reached to 13000 and 50 mbar, respectively. The PCT absorption/desorption kinetics were fitted in real-time by the HTPSwin software, to determine the sufficient equilibration time (the next point would start when the uptake had relaxed just 99% to asymptote). A minimum time of 30 minutes per equilibrium point and a maximum timeout of 300 minutes were set for each kinetic step in both the absorption and desorption isotherms.

## Electronic supplementary material

Supplementary Information
