# Supplementary material for: Synthetic nanocomposite MgH2/5 wt. % TiMn2 powders for solid-hydrogen storage tank integrated with PEM fuel cell
Source: Sci Rep. 2017 Oct 16;7:13296. doi: 10.1038/s41598-017-13483-0 (PMC5643390; doi:10.1038/s41598-017-13483-0)
Supplement: Supplementary file 1 — Supplementary Information [file 41598_2017_13483_MOESM1_ESM.doc]

**Synthetic nanocomposite MgH_2_/5 wt. % TiMn_2_ powders for solid-hydrogen storage tank integrated with PEM fuel cell**

M. Sherif El-Eskandarany ^a^,*, Ehab Shaban^a^, Fahad Aldakheel^a^, Abdullah Alkandary^a^, Montaha Behbehani^b^, M. Al-Saidi^a^

^a^Nanotechnology and Advanced Materials Program, Energy and Building Research Center,

^b^Environment Pollution and Climate Program, Environment and Life Sciences Research Center

Kuwait Institute for Scientific Research, Safat 13109, Kuwait - State of Kuwait

*Correspondence author

Tel.: ‏+(965) 24989265; Fax: +(‏965) 24956609

E-mail address: primary: msherif@kisr.edu.kw

Secondary: msherif99@yahoo.com

Storing hydrogen gas into cylinders under high pressure of 350 bar is not safe and still needs many intensive studies dedicated for tank’s manufacturing. Liquid hydrogen faces also severe practical difficulties due to its very low density, leading to larger fuel tanks three times larger than traditional gasoline tank. Moreover, converting hydrogen gas into liquid phase is not an economic process since it consumes high energy needed to cool down the gas temperature to -252.8 ^o^C. One practical solution is storing hydrogen gas in metal lattice such as Mg powder and its nanocomposites in the form of MgH_2_. There are two major issues should be solved first. One related to MgH_2_ in which its inherent poor hydrogenation / dehydrogenation kinetics and high thermal stability must be improved. Secondly, related to providing a safe tank. Here we have succeeded to prepare a new binary system of MgH_2_/5 wt.% TiMn_2_ nanocomposite powder that show excellent hydrogenation/dehydrogenation behavior at relatively low temperature (250 ^o^C) with long cycle-life-time (1400 h). Moreover, a simple hydrogen storage tank filled with our synthetic nanocomposite powders was designed and tested in electrical charging a battery of a cell phone device at 180 ^o^C through a commercial fuel cell.

*
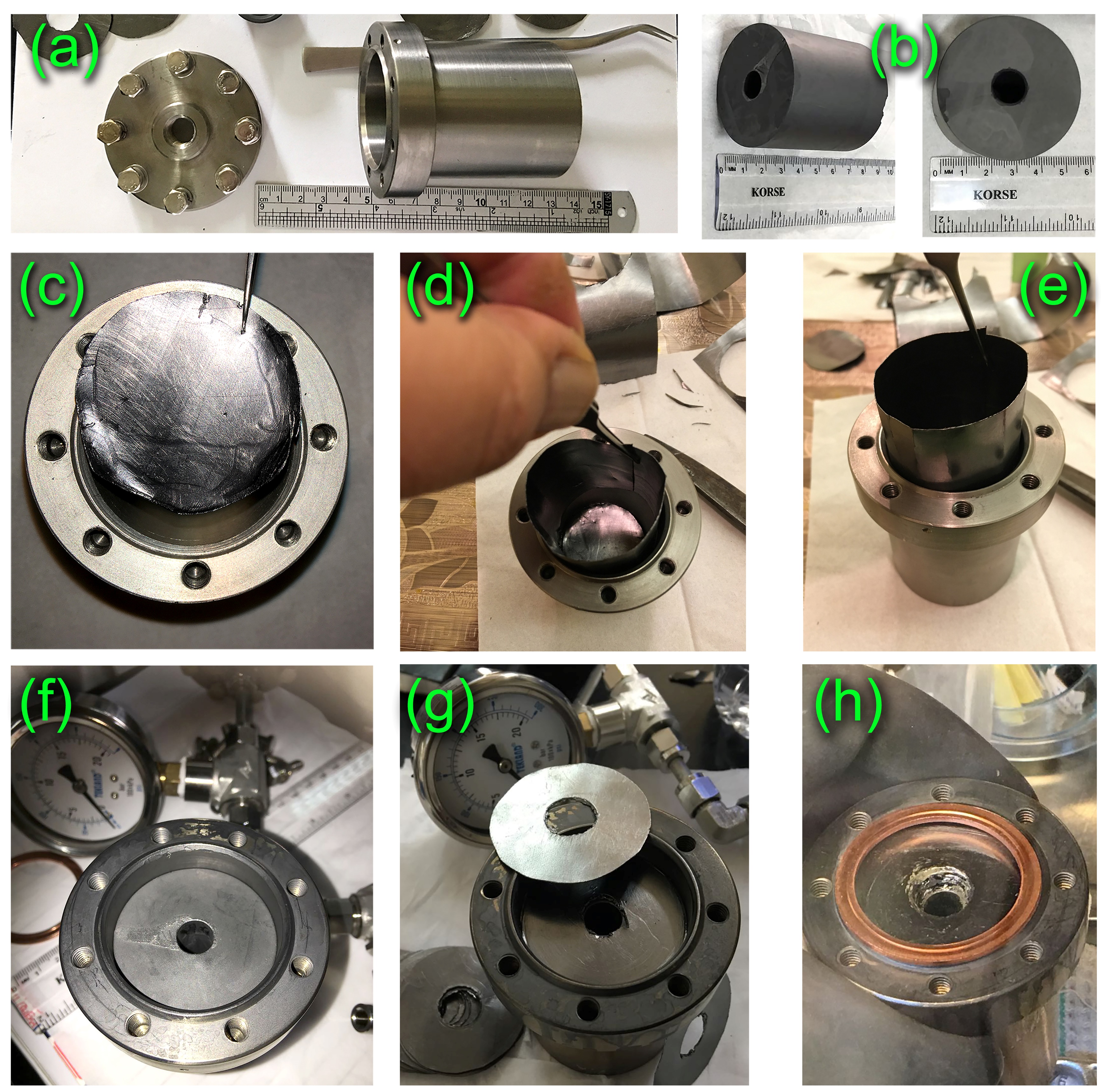
*

**Supplementary Materials, Fig-S1. Raw materials and components used for assembling a prototype of hydrogen storage tank.** (a) elevation view of pure Ti metal hydrogen tank with its 8-screw cap, (b) a graphite die with single vertical hollow feature, (c) solid-graphite bracket-bottom, (d, e) hollow-graphite wall bracket spacer with a diameter of 5.3 cm and 0.3 cm thickness, (f) the graphite die was tightly inserted into the Ti-metal tank, where the MgH2/5 wt.% TiMn_2_ powders were charged into the cylindrical vertical hole, (g) set of graphite spacer having holes in the center were used as top-spacer single, (h) copper metal gasket used for sealing the system.
